# Supplementary material for: Geographic shifts in Aedes aegypti habitat suitability in Ecuador using larval surveillance data and ecological niche modeling: Implications of climate change for public health vector control
Source: PLoS Negl Trop Dis. 2019 Apr 17;13(4):e0007322. doi: 10.1371/journal.pntd.0007322 (PMC6488096; doi:10.1371/journal.pntd.0007322)
Supplement: S1 Table — Each experiment was performed with a randomly chosen subset (75%) of LI presence points. (DOCX) [file pntd.0007322.s001.docx]

**S1 Table.** Accuracy metrics for best model subsets built with the original dataset of aggregated LI occurrence points provided by the MSP using the full set of environmental coverage variables. Each experiment was performed with a randomly chosen subset (75%) of LI presence points.

| Experiment | AUC | Avg. Commission | Avg. Omission |
| --- | --- | --- | --- |
| 1 | 0.66 | 69.49 | 10.88 |
| 2 | 0.68 | 76.09 | 3.41 |
| 3 | 0.61 | 72.72 | 10.54 |
| 4 | 0.63 | 71.23 | 9.67 |
| 5 | 0.69 | 67.93 | 7.58 |
| 6 | 0.63 | 73.95 | 7.58 |
| 7 | 0.65 | 66.45 | 11.31 |
| 8 | 0.63 | 74.94 | 6.04 |
| 9 | 0.63 | 68.33 | 10.65 |
| 10 | 0.65 | 61.18 | 2.97 |
