# Supplementary material for: Geographic shifts in Aedes aegypti habitat suitability in Ecuador using larval surveillance data and ecological niche modeling: Implications of climate change for public health vector control
Source: PLoS Negl Trop Dis. 2019 Apr 17;13(4):e0007322. doi: 10.1371/journal.pntd.0007322 (PMC6488096; doi:10.1371/journal.pntd.0007322)
Supplement: S2 Table — (DOCX) [file pntd.0007322.s002.docx]

**S2 Table.** Prevalence of environmental coverages in model building ruleset.

| Environmental Variable | Ruleset Prevalence |
| --- | --- |
| Elev | 0.94 |
| Bio 5 | 0.94 |
| Bio 7 | 0.91 |
| Bio 8 | 0.82 |
| Bio 9 | 0.85 |
| Bio 10 | 0.85 |
| Bio 11 | 0.74 |
| Bio 13 | 0.88 |
| Bio 15 | 0.68 |
| Bio 17 | 0.94 |
| Bio 19 | 0.85 |
| GPW | 0.74 |
