# Supplementary figures and images for: Geographic shifts in Aedes aegypti habitat suitability in Ecuador using larval surveillance data and ecological niche modeling: Implications of climate change for public health vector control
Source: PLoS Negl Trop Dis. 2019 Apr 17;13(4):e0007322. doi: 10.1371/journal.pntd.0007322 (PMC6488096; doi:10.1371/journal.pntd.0007322)

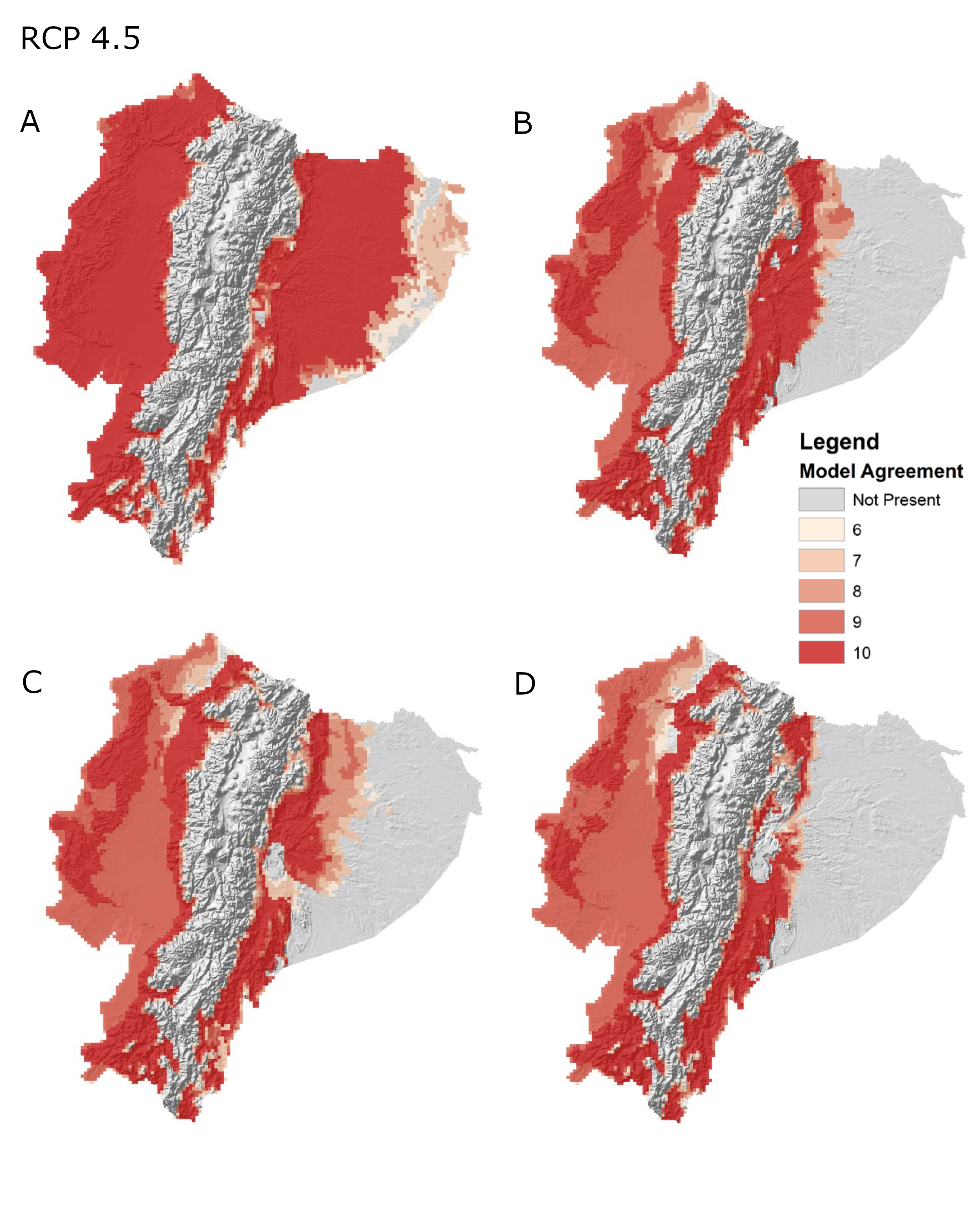

Supplement: S1 Fig — Agreement of best model subsets built with best ranked suite of environmental variables for larval Aedes aegypti presence in Ecuador under A) current climate conditions and future climate conditions projected to the year 2050 under Representative Concentration Pathway (RCP) 4.5 for the B) BCC-CSM-1, C) CCSM4, and D) HADGEM2-ES General Circulation Models (GCM) climate models. This figure was produced in ArcMap 10.4 (ESRI, Redlands, CA) using rasters of model output produced with DesktopGARP (ver. 1.1.3), and elevation data freely available from NASA’s Shuttle Radar Topography Mission (jpl.nasa.gov/srtm). (TIF) [file pntd.0007322.s003.tif]

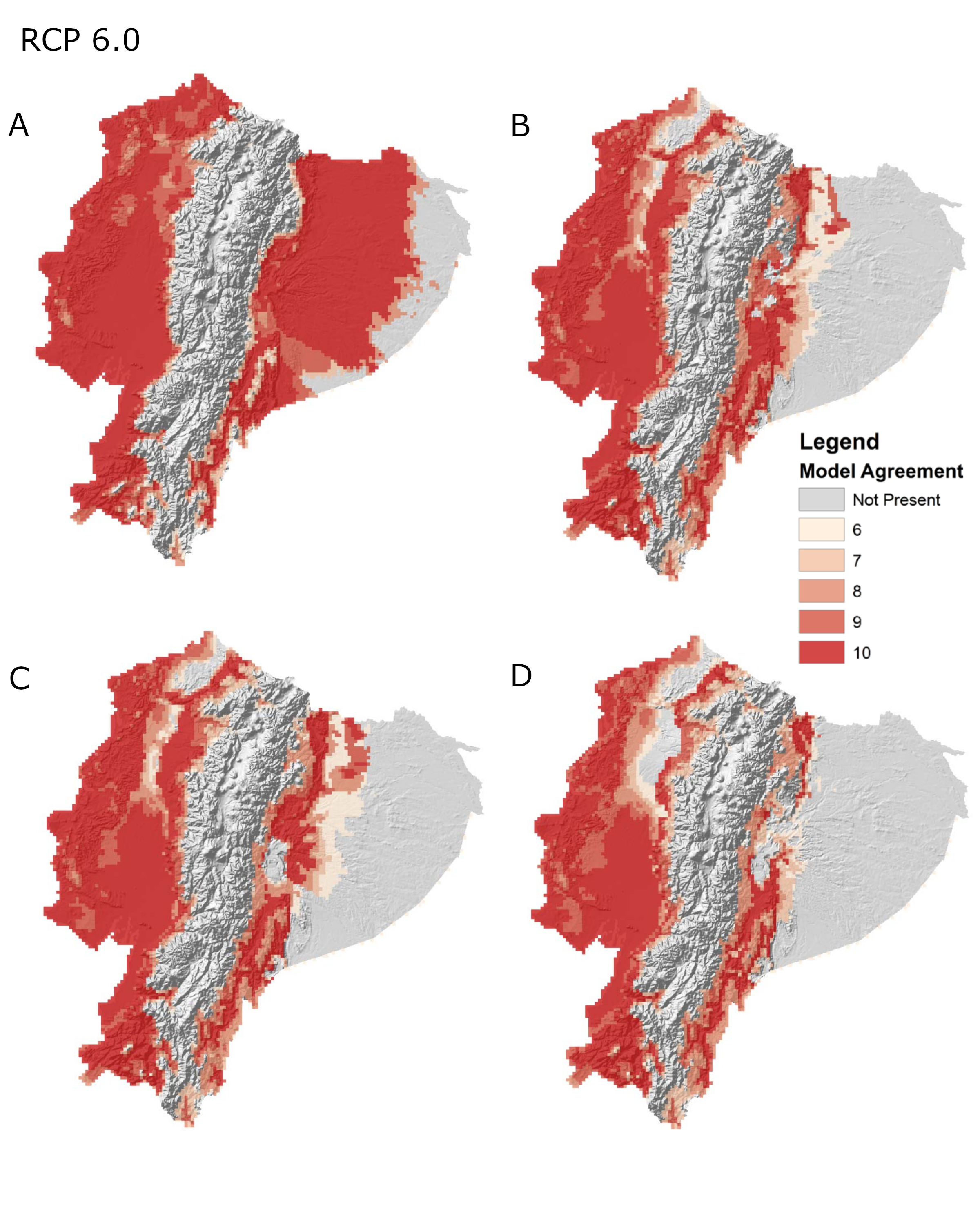

Supplement: S2 Fig — Agreement of best model subsets built with best ranked suite of environmental variables for larval Aedes aegypti presence in Ecuador under A) current climate conditions and future climate conditions projected to the year 2050 under Representative Concentration Pathway (RCP) 6.0 for the B) BCC-CSM-1, C) CCSM4, and D) HADGEM2-ES General Circulation Models (GCM) climate models. This figure was produced in ArcMap 10.4 (ESRI, Redlands, CA) using rasters of model output produced with DesktopGARP (ver. 1.1.3), and elevation data freely available from NASA’s Shuttle Radar Topography Mission (jpl.nasa.gov/srtm). (TIF) [file pntd.0007322.s004.tif]
